# Dust Devil: The Life and Times of the Fungus That Causes Valley Fever

**DOI:** 10.1371/journal.ppat.1004762

**Published:** 2015-05-14

**Authors:** Eric R. G. Lewis, Jolene R. Bowers, Bridget M. Barker

**Affiliations:** 1 Pathogen Genomics Division, Translational Genomics Institute, Flagstaff, Arizona, United States of America; 2 Northern Arizona Center for Valley Fever Research, Translational Genomics Institute, Flagstaff, Arizona, United States of America; 3 Center for Microbial Genetic and Genomics, Department of Biology, Northern Arizona University, Flagstaff, Arizona, United States of America; Geisel School of Medicine at Dartmouth, UNITED STATES

## *Coccidioides* Biology

*Coccidioides immitis* and *C*. *posadasii* are pathogenic, dimorphic, soil-dwelling Ascomycetes in the Onygenales order. On average, both *Coccidioides* species have 29 Mb haploid genomes, containing approximately 10,000 open reading frames (ORFs) on five chromosomes [1]. *Coccidioides*’ most recent common ancestor underwent gene family expansions for proteases and keratinases, membrane biology genes, and toxin production, all likely utilized for survival in animal tissues and morphological changes; and a loss of genes associated with degradation of plant tissue, such as tannases, cellulases, and cutinases [1]. *Coccidioides* and other fungi in the family Onygenaceae are able to degrade keratin and may cause skin disease in humans and animals. Both species of *Coccidioides* are distantly related to other dimorphic human pathogens, such as *Histoplasma (Ajellomyces) capusulatum*, in the new family Ajellomycetaceae [2].

**Fig 1:**
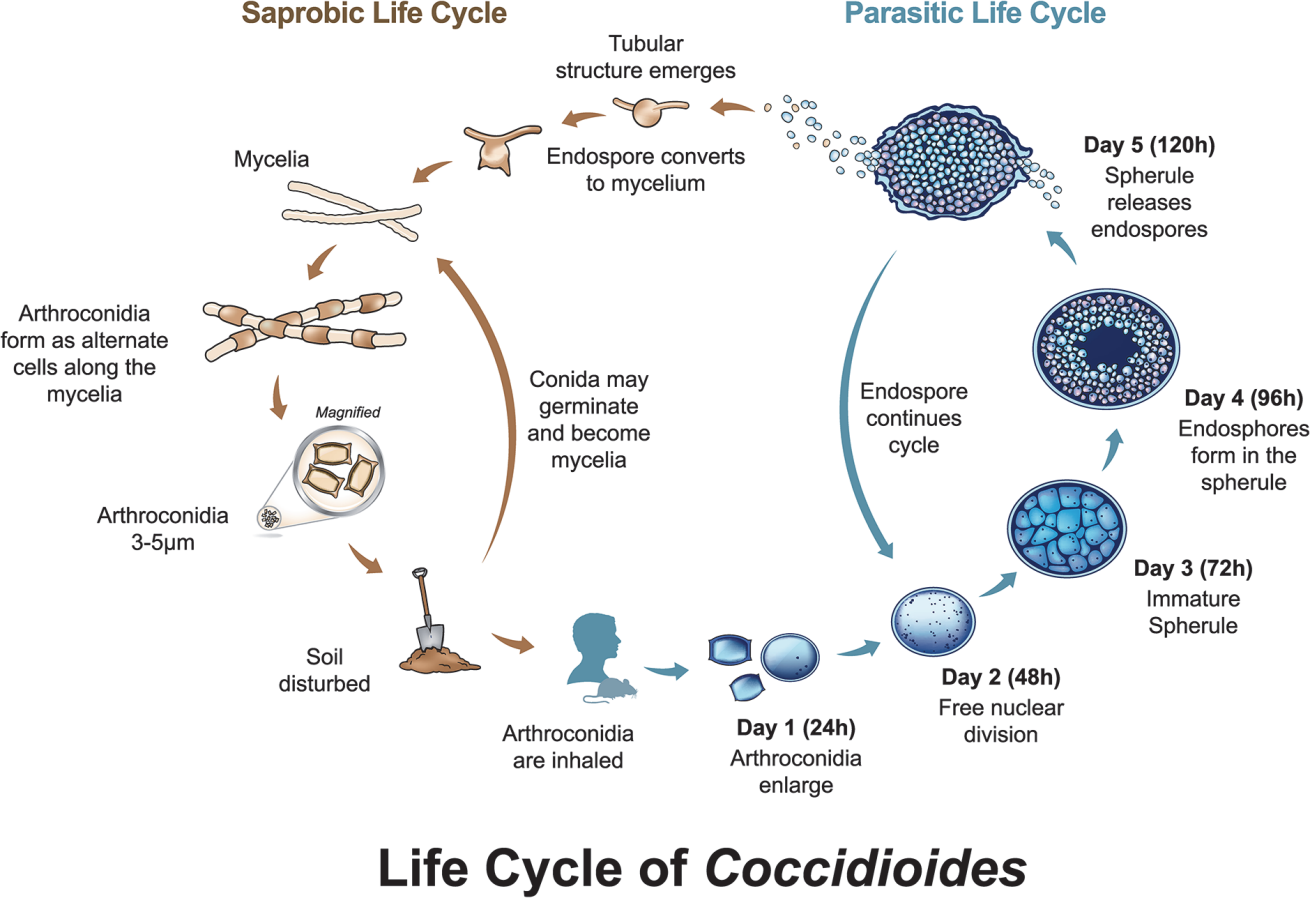
Life cycle of *Coccidioides*. Both *Coccidioides* species share the same asexual life cycle, switching between saprobic (on left) and parasitic (on right) life stages. The saprobic cycle is found in the environment, and produces the infectious arthroconidia. The conidia may be inhaled by a susceptible host, or may return to the environment to continue the saprobic life cycle. The parasitic life cycle is initiated when arthroconidia enlarge and transform into immature spherules, either in vivo or under specific in vitro conditions. From 24 to 72 hours, spherules undergo free nuclear division and begin developing endospores. From 72 to 120 hours, the mature spherules rupture to release endospores. Each endospore can initiate a new spherule, or, under particular atmospheric conditions, nutrient changes, and/or lower temperature, the endospore can convert to a mycelium and initiate the saprobic phase. This occurs in rare circumstances in the living host [45], but is found most commonly in the environment.

Both *Coccidioides* species have similar biology, with a well-characterized asexual life cycle with distinct saprobic and parasitic stages, and only molecular evidence of a sexual cycle (Fig 1). In the saprobic phase, *Coccidioides* cycles between mycelial and arthroconidial stages. Arthroconidia are abscised and become airborne by soil disturbance. Inhalation of arthroconidia by a potential host can lead to coccidioidomycosis, commonly known as (San Joaquin) Valley fever. In an infected host, *Coccidioides* cycles between uninucleate endospores and multinucleate spherules (Fig 1). Differential phenotypes between the species, including temperature sensitivity and salt tolerance, have been described [3] (personal communication, Marc Orbach to B. Barker). No differential disease phenotypes have been investigated, although extreme variation in virulence among strains is documented [4]. The most pathogenic strains can cause fatal disease within eight days with as few as 50 arthroconidia administered intranasally in immunocompetent mice, and some cause much later onset of disease symptoms and death [4–6]. For humans, minimum dosage is not known, but it has been stated that the infectious dose is a single arthroconidium [7]. Both species have been shown to infect a wide variety of mammals, with varying levels of disease [8].

Molecular evidence of mating includes identification and characterization of mating type loci, recombination, and distinct gene genealogies [9–11]. In addition to intra-species sexual recombination, population genomics revealed signatures of hybridization and gene introgression between the two species [12]. At this time, no laboratory-controlled genetic recombination or sexual structures have been described.

## *Coccidioides* Ecology and Population Genetics

The recognition that the genus *Coccidioides* contains two species is a relatively recent discovery [3]. *C*. *immitis* is the name of the first species described, with isolates initially being categorized as California and non-California *C*. *immitis*. Non-California *C*. *immitis* was later found to be another species, *C*. *posadasii* [3]. *C*. *immitis* is found primarily in the San Joaquin Valley of California. However, recent work has also identified this species in Utah and eastern Washington state [13,14]. Whether the fungus has always been present, or if this reflects a more recent migration, is unknown. *C*. *posadasii* is found from Arizona to Texas, and throughout Mexico into Central and South America. Similarity of genotypes from Texas and South America indicates a more recent introduction of *C*. *posadasii* into this region [15]. However, a more complete study of patient and environmental isolates is needed before strong conclusions can be drawn [16]. Indeed, the majority of analyses to date have relied on fungal isolates obtained from human patients, which likely does not represent the overall diversity that occurs in nature.

Multiple analyses of many strains from several geographic locations reveal a high degree of diversity, with little to no clonal structure [3,11,17]. The only nearly identical genome sequences are multiple isolations from the same patient, tracking organ transplant from donor to recipient, and a match of a soil isolate to a patient [13,17,18]. In fact, analysis at a single 10-meter square area in Tucson, Arizona, revealed multiple genotypes present in a single environmental location [11]. Genetic diversity supports the idea that the recent increase in human coccidioidomycosis in the endemic regions is not due to an emerging hyper-virulent strain, but rather an overall increase in exposure of susceptible hosts to environmentally occurring arthroconidia [16,17].

The small number of studies focused on identifying the natural host makes it difficult at this time to make a general statement regarding the ecological niche of *Coccidioides*. As a pathogen infecting mainly mammals, the suggestion has been made that desert rodents, specifically the heteromyids, are the primary host species for *Coccidioides* [1,11,19]. Many North American soils that test positive for *Coccidioides* are associated with rodent burrows or rodent activity [11,20,21]. Studies in South America also found *Coccidioides* associated with armadillos and bats [22,23]. At this time, the natural host and ecology of the organism is not well understood.

## Coccidioidomycosis

Coccidioidomycosis is an endemic disease and *Coccidioides* fungi are biosafety level 3 organisms, just recently removed from the Federal Select Agent list (1995–2013). Based on early studies in which a skin test measured a delayed type hypersensitivity (DTH) to *Coccidioides* antigen to indicate previous infection, approximately 60% of natural human infections are asymptomatic [20]. In the 1990s, the United States discontinued the DTH skin test for clinical use, but recently it has received new U.S. Food and Drug Administration (FDA) approval (FDA approval letter 07/29/2011). As of yet, a comprehensive study of infection rates in the general population in all endemic areas has not been conducted. The primary clinical presentation of coccidioidomycosis is pneumonia, which generally resolves without treatment. Some hosts will carry lung nodules or cavities of viable *Coccidioides*; however, incidence and long-term consequences of asymptomatic carriage are unknown. Others suffer chronic disease, sometimes requiring life-long antifungal treatment. Infections disseminate in fewer than 1% of cases, and create lesions at a single body site or potentially affect multiple organ systems. Central nervous system involvement is often fatal if left untreated [20]. Coccidioidomycosis is diagnosed by serology, microscopy, antigen detection, and/or culture, all of which have limitations [24,25].

Cellular immunity, generated by a Th1 response and manifested by DTH, is essential to defense against coccidioidomycosis and long-lived protection from reinfection [26]. Several studies show that response to *Coccidioides* is more effective in the presence of immune factors such as interferon gamma (IFNγ) released by Th1 cells [27]. Recent data substantiate an essential role for Th17 pathway induction in mice for long-term immunity as well [28]. Patients with chronic disease appear to mount a non-protective Th2-type humoral immune response [27]. Immunodeficiency, either genetic or acquired, is a major risk factor for disseminated disease [29,30].

Many known and unknown factors shape the immune response to *Coccidioides* and manifestation of disease. For reasons that are not well understood, African Americans and Filipinos appear at a greater risk for disseminated coccidioidomycosis than other ethnicities [27,31]. Underlying health issues and elder age are also known risk factors for more severe disease [20]. Different strains of *Coccidioides* affect the magnitude of the immediate immune response [27,32]. Few studies have elucidated roles of other important cell types, such as natural killer cells or dendritic cells, which produce IFNγ to activate macrophages and IL-12 to activate T cells. The current data suggest that a complex combination of host immune factors determines the advancement or clearance of infection [26].

## Initial Stages of Infection

The largest gap in our knowledge of host immunity is the first five days of the innate response. The initial encounter of inhaled arthroconidia with the host lung is not well understood. It is believed that arthroconidia reach the alveoli where mucociliary clearance factors are encountered. Lung epithelial cells are equipped with pattern recognition receptors (PRRs), such as toll-like receptors (TLRs) and Dectin-1, which are capable of inducing immediate effector responses [33], and these cells influence alveolar macrophage regulation [34]. Yet, the role of epithelial cells in response to *Coccidioides* inhalation is unknown.

In the lungs, innate immune cells recognize fungal components using multiple receptors, inducing phagocytosis and production of reactive oxygen species (ROS). Resident alveolar macrophages are naturally tolerant to prevent overreaction, with low phagocytic activity and respiratory burst [34]. Upon encounter with arthroconidia, non-fungicidal macrophage engulfment occurs. Within hours, there is a *Coccidioides*-antigen activated influx of polymorphonuclear neutrophils (PMNs) [35], which may enhance spherule formation [36]. PMNs respond in a similar manner as macrophages, engulfing arthroconidia without killing them [37]. Direct observation implicates failure of phagosome-lysosome fusion, relieving the arthroconidia from contact with lytic enzymes. When activated in vitro with IFNγ or T cells from an immune host, the phagocytes’ ability to kill arthroconidia increases [37,38]. This may be a contributing factor to the success of IFNγ as a therapy for disseminated coccidioidomycosis [39].

Based on in vitro and in vivo observations, arthroconidia enlarge and transform into immature spherules (Fig 1, parasitic life cycle). In addition to the induction of phagocytosis and ROS production, TLR-2 and Dectin-1, when bound by spherules, collaborate to trigger cascades mediated by MyD88 and Card-9 intracellular adaptors [40]. Subsequent activation of transcription factor NFκB produces proinflammatory cytokines TNFα, MIP-2, and IL-6, which are essential effectors of Th1 and Th17 cellular responses [40,41]. Dectin-1 also mediates production of critical Th1 cytokines IL-12 and IFNγ, and Th17 cytokines IL-23, IL-17a, IL-22, and IL-1β [41]. Spherules increase mRNA expression and other factors for evasion of host Dectin-1, as well as resistance to, and suppression of, host oxidative defenses [42].

For the next few days (days two to three), spherules undergo free nuclear division, and mature into large (30 to 80μm) septate cells containing developing endospores. Some literature suggests that growing intracellular spherules lyse their phagocytes, but direct evidence is lacking. Regardless, spherules are probably too large for phagocytosis. Additionally, spherules produce an alkaline extracellular matrix (ECM) that prevents PMN contact and degranulation-induced damage [32,43].

On day four or five, the mature spherules rupture to release endospores, although this process may take longer in vitro. A renewed host response comprises another influx of PMNs. PMNs and macrophages readily engulf the endospores. However, phagocytosis is still impeded as endospores can remain in large clusters tied by fibrillar structures originating from the spherule outer wall [37] and are protected by the spherule ECM [32,43]. From this point, the *Coccidioides* parasitic cycle continues (Fig 1). At this time, host and/or pathogen mechanisms terminating this cycle in the majority of hosts are unknown.

## Future Directions

*Coccidioides* species and the mycoses they cause have been studied for over a hundred years. With this review, we attempt to summarize some of the more recent findings that have taken place in this field of research. Nevertheless, there is still a dearth of information about these fungal organisms and how they survive within the environment and inside host organisms.

Multiple research aims can greatly advance this research field. Analyzing environmental *Coccidioides* isolates would increase our understanding of the ecology of these fungi. Further characterizing host and pathogen genetics that influence the course of disease could lead to a better understanding of mycosis and development of immunotherapies for disease. Screening *Coccidioides* for susceptibility to novel therapeutics, particularly those that are being developed for other fungi, will aid in developing new treatments to eliminate the fungus from the patient to prevent reactivation of disease.

Many of the previous immunological studies employed various in vitro methodologies to answer scientific questions while adhering to the biosafety regulations that governed *Coccidioides* research at the time. Though these studies provided the vital foundation of our current knowledge of *Coccidioides* immunology, the answers provided by those studies may not entirely capture in vivo or in situ events. Previous studies that examined potential vaccine candidates in murine models of coccidioidomycosis were also very beneficial to our understanding of coccidioidomycosis and vaccinology [44]; however, our knowledge of a naïve host immune response to infection remains limited. Now that *Coccidioides* is no longer a Select Agent, and-omic technologies are cheaper and more efficient than ever, the time has come to study the in vivo and in situ host immune response to coccidioidomycosis.
